# Herbivore kairomones affect germination speed, seedling growth, and herbivory

**DOI:** 10.1007/s00442-024-05621-z

**Published:** 2024-09-28

**Authors:** Brooke A. Pellegrini, Lina S. Pintado, Paige N. Souza, Santhi P. Bhavanam, Colin M. Orians, John L. Orrock, Evan L. Preisser

**Affiliations:** 1 Department of Biological Sciences, University of Rhode Island, Kingston, RI 02881 USA; 2 Department of Biology, Tufts University, Medford, MA USA; 3 Department of Integrative Biology, University of Wisconsin, Madison, WI USA

**Keywords:** Herbivory, Kairomone, Germination, Plant–insect, Mollusk, Risk

## Abstract

Seeds and seedlings are particularly vulnerable to herbivory. Unlike mature plants, which can wait until herbivory is experienced to induce defense, seeds and seedlings face mortality if they wait. Slug mucus functions as a kairomone, a non-attack-related substance emitted by consumers that is detected by a prey species (in this case, plants). While snail mucus has been shown to induce defense in seedlings, it is not widely confirmed whether slugs have the same effect and whether seeds can also detect and react to such herbivore cues. We investigated how exposure to *Arion subfuscus* mucus affected growth and defense in *Brassica nigra* seeds and seedlings. Seeds exposed to slug mucus germinated 5% faster than control (water only) seeds, but the resulting seedlings weighed 16% less than control seedlings. To test whether this difference results from herbivore-exposed plants allocating energy from growth to defense, we conducted choice bioassays assessing slug preference for control seedlings versus seedlings that were either (A) exposed to mucus only as a seed; or (B) exposed to mucus as a seed and seedling. While slugs did not differentiate between control seedlings and ones exposed to herbivore cues only as a seed, they ate 88% less biomass of seedlings exposed to mucus as both seeds and seedlings. These results suggest that slug mucus induces changes in plant traits related to defense and growth/competitive ability. Future research should determine the chemical mechanisms of this induced defense.

## Introduction

The structural redundancy and compartmentalized nature of large plants means that even chronic herbivory often poses little threat to their survival or reproduction (Sheriff et al. 2020). As a result, plants generally wait until consumer attack begins before inducing defense (Karban and Baldwin 1997). Such defensive induction is found throughout the plant kingdom and can be triggered by a diverse array of cues: herbivory, exposure to volatile compounds produced by damaged plants (Heil and Karban 2010), herbivore oviposition (Peiffer et al. 2009), and even herbivore saliva (Felton 2008). Reliance on direct damage-related cues (i.e., herbivory) allows plants to avoid inducing energetically costly processes in the absence of a real threat (Huot et al. 2014). This is important, because increased allocation to defense often comes at the cost of reduced growth, meaning that plants that defend themselves unnecessarily may do worse in competition with other plants or have difficulty acquiring nutrients (Orrock et al. 2015). Induced defenses are important to understand, because they are ubiquitous, can ultimately shape plant survival and reproduction and have effects that cascade to other plant and animal species (Karban and Baldwin 1997), and can also be essential for predicting plant response to climate change (Orrock et al. 2018).

Despite the ubiquitous nature of plant-induced defenses, recent studies highlight that our knowledge of the cues that plants use to initiate defense may be incomplete: plants may respond to incidental chemical cues produced by herbivores that are not associated with attack (Helms et al. 2013; Orrock et al. 2018). These chemicals, known as kairomones, can induce changes in anti-predator behavior in animal prey, where kairomone-mediated changes in behavior are often a fundamental component of the ‘ecology of fear’ (Schoeppner and Relyea 2009). The study of kairomones is a cornerstone of predator–prey ecology, and a deeper understanding of the role kairomones play in plant–herbivore interactions may provide greater insight into plant responses to threats.

The general ability of plants to tolerate herbivory belies the vulnerability of both seeds and seedlings. Because plants in these early life stages have minimal energetic reserves, they have a much harder time compensating for tissue loss or damage. Young plants may thus benefit from the ability to detect and respond to pre-attack herbivore cues in a manner similar to heterotrophs, accepting a greater risk of unnecessary defensive induction to reduce the likelihood of damage or death (Orrock et al. 2015; Sheriff et al. 2020). In finding that plant defenses are induced by kairomones, several important questions arise, including questions about what plant life stages respond to kairomones and how plants respond to kairomones. At present, the degree to which kairomone use is common in plants is affected by their recent discovery and the limited number of studies that have been done.

Seed/seedling use of herbivore kairomones to preemptively induce defense was first reported in research demonstrating that locomotion mucus of the snail *Helix aspersa* decreased both growth and herbivory in mucus-exposed *Brassica nigra* seedlings (Orrock 2013). Other studies have shown that terrestrial slug mucus induces jasmonic-acid mediated defense in *Arabidopsis thaliana* (Falk et al. 2014). Both snails and slugs are important herbivores of seeds (Meldau et al. 2014; Miczajka et al. 2019) and seedlings (Buschmann et al. 2006; Fenner et al. 1999; Hanley et al. 2011; Moshgani et al. 2014), and exposure to their locomotion mucus has been shown to affect growth and subsequent herbivore damage in several plant species (Orrock et al. 2018; Zolovs et al. 2022). Although these studies suggest an important and unappreciated role of mollusk kairomones in shaping plant defense and fitness, it is unclear whether seed response to mollusk kairomones is a widespread phenomenon. Moreover, although plant-induced defenses can have significant costs (e.g., reduced growth and competitive ability because plants are investing in defense), it is unclear whether kairomone-mediated plant defenses lead to changes in plant competitive ability.

We hypothesized that both seeds and seedlings will respond to mucus in ways that reduce their likelihood of subsequent herbivore attack. We used complementary factorial experiments to explore whether an annual forb species (*B. nigra*) generates induced defenses when exposed to the kairomone of a terrestrial mollusk (locomotion mucus of *Arion subfuscus*). This system is optimal for exploring kairomone-mediated defense, because *B. nigra* has been shown to respond to snail mucus (Orrock 2013) and it and other brassicaceous species are commonly attacked by both snails and slugs (Bischoff and Tremulot 2011; Buschmann et al. 2006; La Pierre et al. 2010). We quantify seed traits that may be indicative of competitive success (germination speed), use competition assays to directly quantify the potential costs of defense on plant growth, and employ herbivory assays on cotyledons to provide an ecologically relevant measure of the effectiveness of kairomone-mediated defense.

## Materials and methods

### Study species

*Arion subfuscus* was introduced from Europe to North America in the early nineteenth century (Chichester and Getz 1973). It is a polyphagous herbivore that has been described as “having the greatest impact upon natural communities” of any northeastern U.S. terrestrial mollusk (Chichester and Getz 1969) and is the most abundant slug in New England (French 2012). Adults were collected from fallen logs at a forest edge in South Kingstown, RI (USA), in April–September 2022 for the seed germination assays and May–June 2023 for the seed/seedling experiment. Slugs were maintained in the lab on a diet of iceberg lettuce (*Lactuca sativa* var. capitata) and leaf litter in 21–24° C terrariums that were regularly misted to maintain humidity.

*Brassica nigra* is an herbaceous plant native to the Mediterranean and grown globally. Seeds were sourced from Outsidepride Seed Source, LLC (Independence OR USA); *A. subfuscus* is also found in Oregon (Burke 2013). Seeds were kept in a dark drawer, away from sunlight, prior to the experiment. *B. nigra* seeds came in two distinct color morphs, orange and black; we used only black morph seeds in our work to facilitate the visual assessment of germination.

### Germination assays (2022)

Starting in April 2022, we conducted five replicate experiments assessing whether exposure to *A. subfuscus* mucus affected *B. nigra* germination speed (time to germination) and rate (percent of seeds that germinate). Each experiment was conducted in a series of 90 mm petri plates lined with Whatman grade 598 qualitative 90 mm white filter paper (Millipore Sigma, Burlington MA USA) and wet with 2 mL distilled water. Petri plates assigned to the mucus treatment had one adult slug added to each of them, while petri plates assigned to the control treatment had water only. Once the slugs were added, both slug and control plates were interspersed and held in a dark cabinet at 20–24 °C.

After 24 h, all slugs were removed and returned to their terrariums. Once the slugs were removed, a piece of Ahlstrom-Munksjö black qualitative grade 8613 90 mm filter paper (Ahlstrom-Munksjö Paper, Jönköping Barnarpsgatan Sweden) was placed in each petri plate over the white filter paper and wet with 2 mL distilled water. The black color of the filter paper facilitated visual assessment of when the white radicle emerged from each seed. Ten *B. nigra* seeds were then placed on top of the black filter paper in each dish. The mucus and control dishes were then interspersed and held in a dark cabinet at ambient temperature. Depending on the experiment, each petri plate was checked either twice (9 am and 9 pm; April experiment) or three times (9 am, 3 pm, and 9 pm; May, October, and December experiments) daily for seed germination. Germinated was recorded as the number of new germinated seeds in each petri dish since the last check. The checks continued for 5–6 days until all seeds had germinated or germination had ceased. The assay was performed a total of four times. The April experiment had 20 petri plate replicates per treatment, the May experiment had 40 replicates per treatment, the October experiment had 30 replicates per treatment, and the December experiment had 20 replicates per treatment.

### Seed and seedling experiment (2023) (Supplemental Fig. 1)

**Fig. 1:**
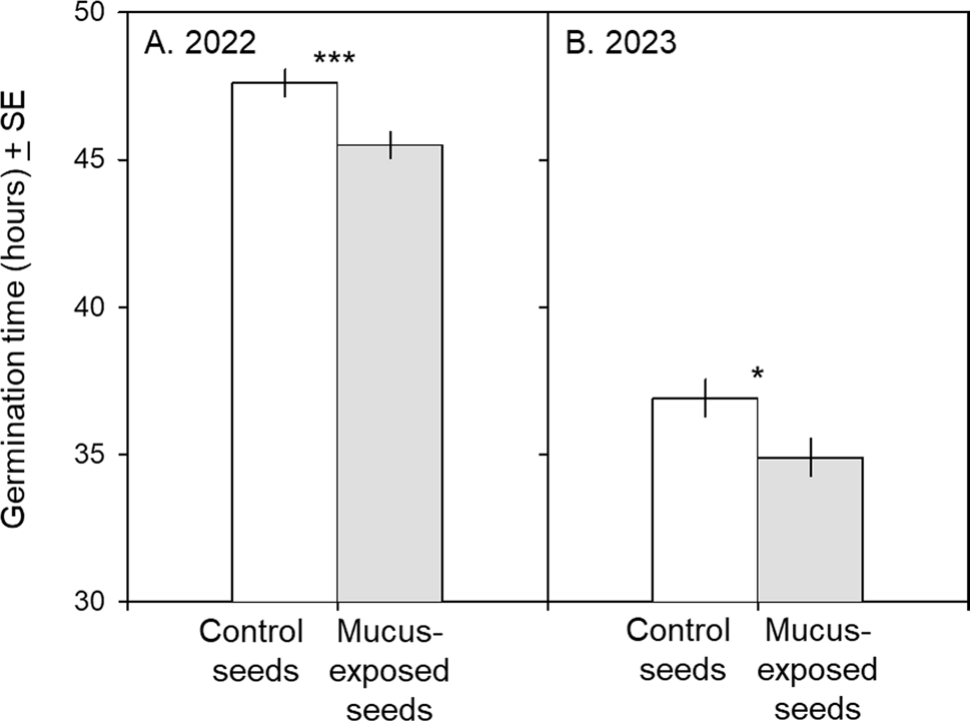
Effect of exposing *B. nigra* seeds to water (white bars) or water and *A. subfuscus* locomotion mucus (gray bars) on germination speed in 2022 (Fig. 1A) and 2023 (Fig. 1B). In both years, mucus-exposed seeds germinated significantly faster than control seeds. *p < 0.05; ***p < 0.001

Prior to the start of the experiment, 130 2.5 × 2.5 cm Styrofoam pots (Carolina Biological Supply Company, Burlington NC USA) were filled with MiracleGro moisture control potting mix (Scotts-MiracleGro Inc., Marysville OH USA). The pots were placed over a mesh screen to allow drainage and prevent pots from being exposed to water draining from other pots.

### Germination assay

The 2023 germination assay portion of the seed and seedling experiment was conducted identically to the 2022 assays except as follows. Each petri plate in the mucus treatment had two adult slugs added to it, after which all petri plates were kept in the dark. There were ten control petri plates and ten mucus petri plates. After 24 h the slugs were removed and 20 *B. nigra* seeds were placed in each control and treatment petri plate. Eighteen hours after the seeds were added to the petri plates, and prior to any germination, three randomly selected seeds were removed from each of the 20 petri plates for use in the competition assay (described below). Starting 24 h after the *B. nigra* seeds were added and continuing every 6 h afterward, the 17 seeds remaining in each plate were checked for germination (defined as a visible radicle). At each check, the number of seeds germinated since the last check in each petri dish was recorded. The initial 24-h sampling point was determined using data from the 2022 assays that checked *B. nigra* germination at 16, 18, 20, and 24 h and found essentially no germination at the first three time points (6 of 2482 germinated seeds; 0.24% of total). Plates were checked until the first ten of seventeen seeds germinated in each petri plate (72 h post).

### Competition assay

In each of 30 Styrofoam pots, a randomly selected seed from the control treatment was planted in one corner and a seed from the mucus treatment was planted in the opposite corner (a total of two seeds per pot). Each side of the pot was labeled with its treatment and originating petri plates. Pots were watered and rotated as described below.

After 3 weeks, the two plants in each pot were carefully removed and rinsed to remove excess soil and separate their root systems. After the plants were soil-free, they were patted dry. Total fresh biomass was measured, and then, the plant was sectioned to measure total above- and belowground fresh biomass.

### ‘Seed exposure’ and ‘seed and seedling exposure’ treatments

The first ten germinated seeds in each dish were each planted in individual Styrofoam pots filled with potting mix and watered to soil saturation beforehand. Pots were placed in front of a window for sunlight, watered every 12 h, and turned 90° each day to ensure even sunlight exposure.

One week after the start of the experiment, half of the seedlings grown from mucus-treated seeds were assigned to receive additional mucus exposure (‘seed and seedling exposure’); the other half of the seedlings were not exposed (‘seed exposure’).

To generate the additional slime cue for each ‘seed and seedling exposure’ pot, two *A. subfuscus* were placed into a petri plate containing 0.32 g sifted (425 μm sieve) potting mix wetted with 1.3 mL distilled water. After 24 h, the slugs were removed, and the soil was mixed to evenly incorporate the slime into the soil. A 0.3 g aliquot of mucus-containing soil was then placed on top of the existing soil in a ‘seed and seedling exposure’ pot and the pot watered immediately afterward. This procedure was repeated for each ‘seed and seedling exposure’ pot on days 7, 9, and 12 after planting, with new petri plates and slugs used for each application.

Seedlings in the ‘seed exposure’ and ‘control’ treatments did not receive additional soil during growth. To control for the effect of soil addition, we added a fourth ‘control and soil’ control treatment in which a set of control plants received 0.3 g aliquots of wetted no-mucus soil on top of the existing soil on days 7, 9, and 12. All plants were watered as above for a total of 3 weeks after planting.

### Slug choice bioassay

Three weeks after the start of the experiment, choice bioassays were done in which *A. subfuscus* were allowed to choose between a cotyledon from a ‘control’ plant and one from either a A) ‘seed exposure’ plant or B) ‘seed and seedling exposure’ plant. There were 15 replicates for the ‘control’ versus ‘seed exposure’ comparison, and 17 replicates for the ‘control and soil’ versus ‘seed and seedling exposure’ comparison.

For each bioassay, a cotyledon was cut from a seedling and its fresh weight recorded; we attempted whenever possible to pair similarly sized cotyledons. A 90 mm petri plate was sprayed once with distilled water using a perfume atomizer before a control and treatment cotyledon were placed next to each other on the left and right halves of the dish. The control cotyledon was placed on the left side in odd-numbered replicates and the right side in even-numbered replicates.

One day prior to the start of the bioassays, mature *A. subfuscus* were removed from their terrarium and held individually in a wetted petri plate. After being starved for 24 h, each slug was weighed and then placed in the middle of the petri plate equidistant from the two cotyledons. The petri plate was then closed, and each slug allowed to forage. After 2 h, the slug was removed, and both cotyledons reweighed. We compared the amount of cotyledon tissue consumed as a measure of slug preference. Data from replicates in which the slug did not feed on either cotyledon were excluded from analysis.

### Statistical methods

#### Germination assays

Because the germination assays in 2022 used slightly different methods than those in 2023, we analyzed them separately (although we note that combining them does not alter our conclusions). To compare differences in the proportion of seeds in each petri plate that germinated, we used a generalized linear mixed model with a binomial response distribution. To evaluate the effect of slug mucus on the rate of germination, we used survival analysis (McNair et al. 2012). We used restricted mean survival time (Zhao et al. 2015), because it is more robust in situations where treatments have strong time-dependent treatment effects (i.e., situations that can violate the proportional hazards assumption of Cox proportional hazards regression; Zhao et al. 2015). An additional benefit of restricted mean survival time is that it produces estimates over a defined period that is relevant for ecological hypotheses (whereas Cox regression produces ratios of hazard functions that may be less intuitive). Changes in germination timing that might arise due to mucus might be expected to change the degree to which seedlings experience competition, because seeds that germinate earlier experience the benefits of being able to grow before other seeds germinate (e.g., Orrock and Christopher 2010). As such, a relevant ecological period over which to evaluate germination is the amount of time that it takes the majority of seeds to germinate (and hence the window of time when germinating early can yield benefits). We define the evaluation period as the time when > 80% of seeds had germinated; this period was 72 h for the 2022 experiments and 48 h for the 2023 experiment.

#### Competition assay

We used a general linear mixed model to examine the effect of mucus exposure on the growth of individual plants grown in competitive conditions. The dependent variable we examined was total plant biomass (the sum of above- and belowground biomass). The model included a fixed effect of treatment (no-mucus control vs. exposure to mucus as a seed and seedling). The model included random effects to account for variation due to seeds experiencing the same conditions within a petri plate. The model also included a random effect to accommodate variation arising due to the unique pair of plants grown together in each replicate pot, reflecting the paired nature of our experimental design (i.e., two plants with different treatments were grown in the same pot).

#### Slug choice bioassay

We examined the effect of mucus exposure on slug herbivory by comparing the proportion of plant mass remaining following the herbivory trial using linear mixed effect models. Because of slight differences in methodology, we conducted separate analyses for plants exposed as seeds and a separate analysis for plants exposed both as seeds and as seedlings. Because our assays used a paired design that presented slugs with tissue from an untreated (control) and a mucus-treated plant, we used the treatment arena as a random effect (analogous to a paired t test).

Statistical analyses were carried out using R 4.3.2 (R Core Team 2023), the lme4 package (Bates et al. 2015), and the survRM2 package (Uno et al. 2022).

## Results

### Germination assays (2022)

Mucus-exposed seeds germinated more quickly than control seeds (45.5 ± 0.45 SE hours and 47.6 ± 0.46 h, respectively; likelihood-ratio test from RMST, χ^2^_1df_ = 10.83, *p* = 0.001; Fig. 1A). Germination rate was high for both mucus-exposed and control seeds (97.2 ± 2.3% SE and 97.2 ± 2.0%, respectively) and did not differ between treatments (χ^2^_1df_ = 0.34, *p* = 0.55).

### Seed and seedling experiment (2023)

#### Seed germination

Again, mucus-exposed seeds germinated more quickly than control seeds (34.9 ± 0.63 SE hours and 36.9 ± 0.63 h, respectively; likelihood-ratio test from RMST, χ^2^_1df_ = 5.17, *p* = 0.023; Fig. 1B). Germination rate was high for both mucus-exposed and control seeds (97.6 ± 1.2% SE and 96.5 ± 1.4%, respectively) and did not differ between treatments (χ^2^_1df_ = 0.41, *p* = 0.52).

#### Seedling competition

**Fig. 2:**
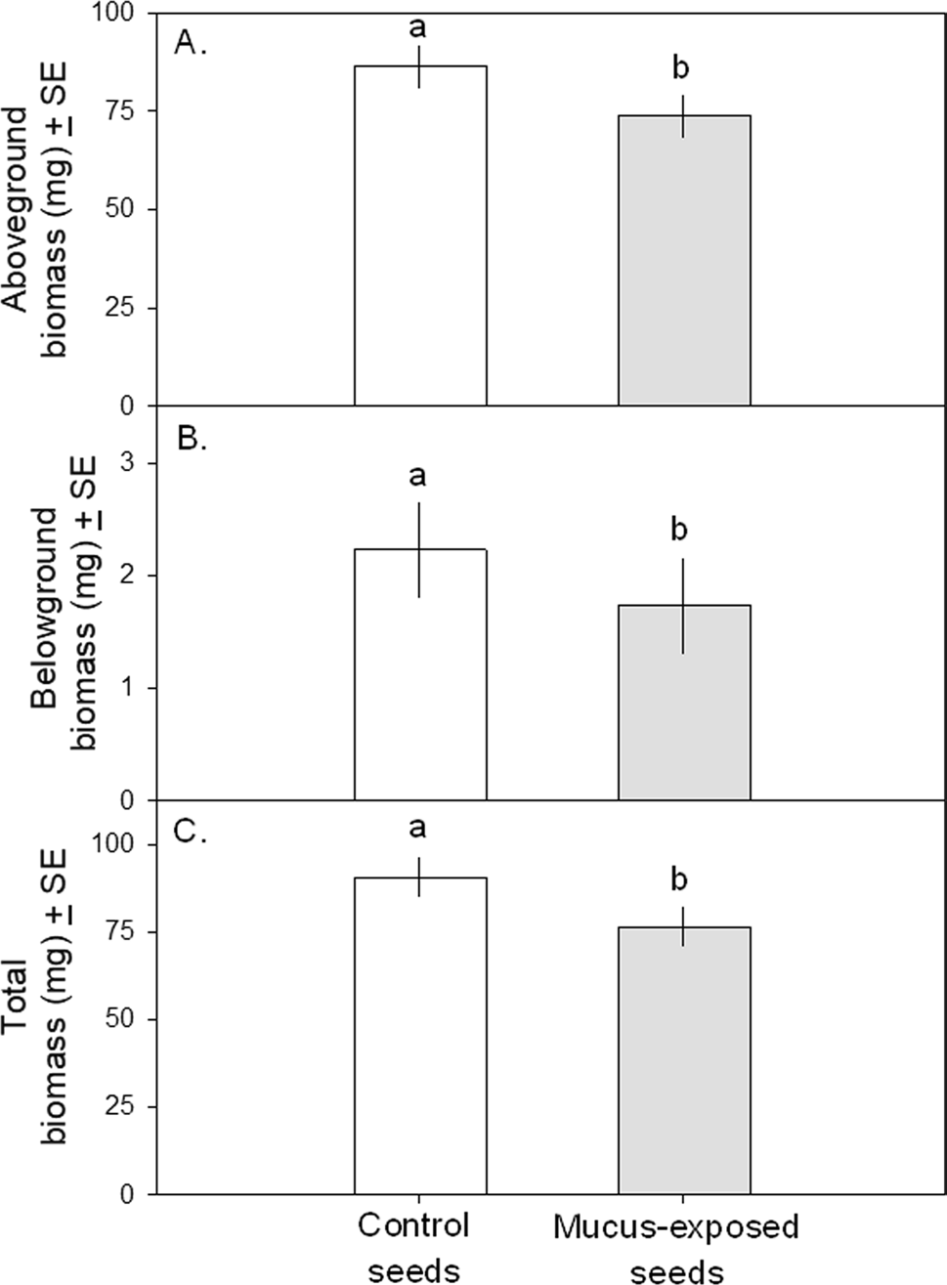
Effect of exposing *B. nigra* seeds to water or *A. subfuscus* locomotion mucus on seedling aboveground (Fig. 2A), belowground (Fig. 2B), and total (Fig. 2C) biomass. Seedlings from mucus-exposed seeds had significantly lower aboveground, belowground, and total biomass than seedlings from control seeds. Lowercase letters denote significant differences at α = 0.05 (Tukey’s HSD)

After 3 weeks, seedlings grown from mucus-exposed seeds were smaller than control seedlings (Fig. 2). Aboveground and belowground biomass were tightly correlated (r = 0.50, t_48_ = 3.98, *p* = 0.0002), and treatment-level differences were apparent in both aboveground (Fig. 2A; F_1,24.6_ = 6.19, *p* = 0.020) and belowground (Fig. 2B; F_1,23.9_ = 4.62, *p* = 0.042) measures. This resulted in mucus-exposed seedlings weighing 16% less than controls (Fig. 2C; F_1,25.0_ = 8.25, *p* = 0.008).

#### Slug choice bioassay

**Fig. 3:**
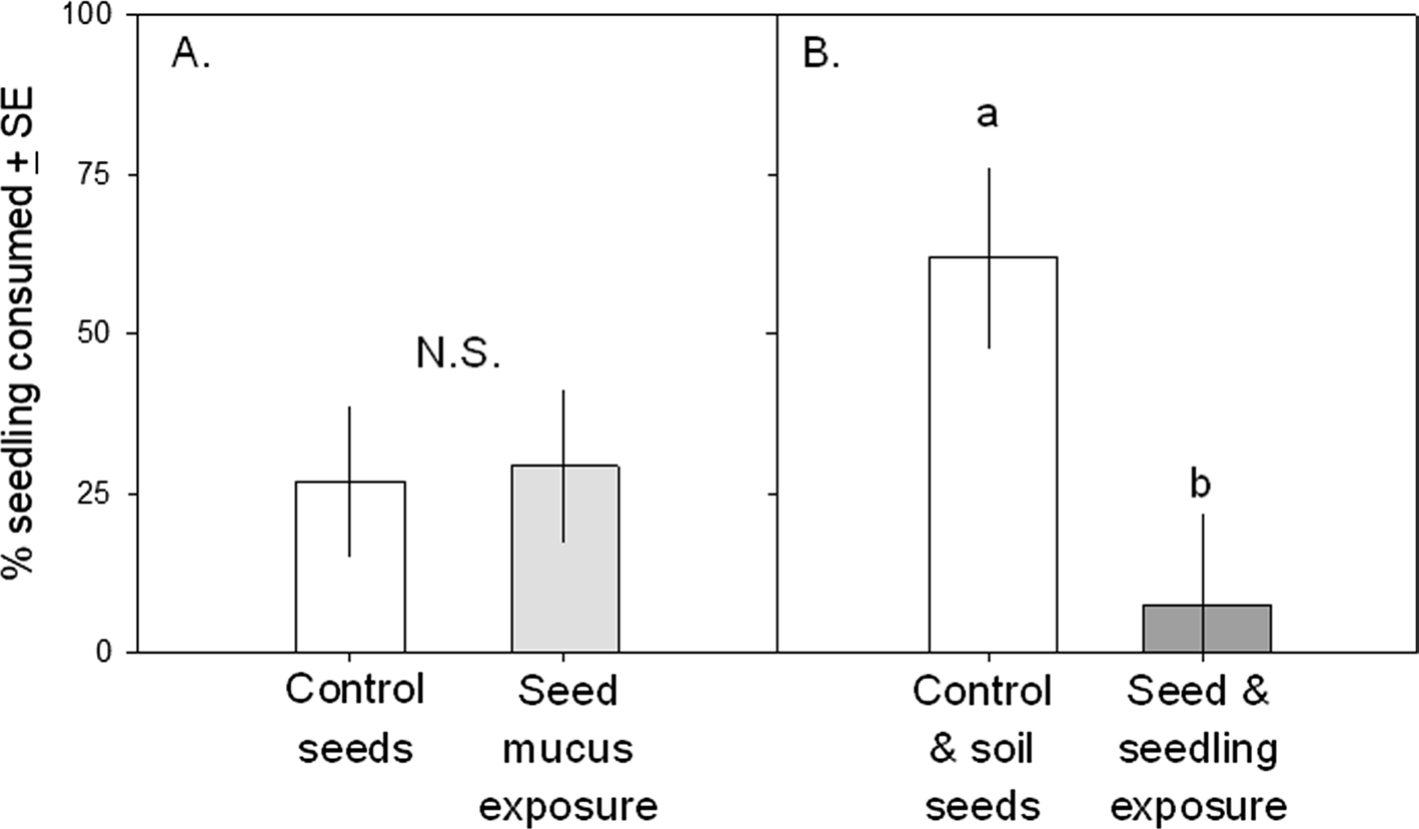
Proportion of seedlings eaten by *A. subfuscus* allowed to choose between seedlings from the four treatments. 3**A**: control (exposed to water as seeds) versus treatment (exposed to *A. subfuscus* mucus as seeds only) seedlings; 3**B**: control (exposed to water as seeds and water-treated soil as seedlings) versus treatment (exposed to *A. subfuscus* mucus as seeds and mucus-treated soil as seedlings) seedlings. Slugs did not differentiate between control plants and plants grown from mucus-treated seeds but did prefer control plants over those grown from mucus-treated seeds and exposed to mucus-treated soil as seedlings. Lowercase letters denote significant differences at α = 0.05 (Tukey’s HSD); *N.S.* not significant

*Arion subfuscus* did not differentiate between cotyledons grown from mucus-exposed seeds and control seedlings (Fig. 3A; F_1,12_ = 0.02, *p* = 0.89). They did, however, prefer control and soil plants over seedlings exposed to mucus at both the seed and seedling stage (Fig. 3B, F1,12 = 7.34, *p* = 0.019).

## Discussion

Cues of attack risk are essential to induce the plant defenses vital for preserving fitness. For example, non-chemical cues such as vibrations from chewing increased glucosinolate and anthocyanin levels in *Arabidopsis thaliana*, indicating a defense response (Appel et al. 2014). However, the degree to which plants detect and respond to herbivore cues as indicators of risk has only recently become a topic of study (Helms et al. 2017; Meldau et al. 2014; Orrock et al. 2018). Our finding that *B. nigra* seeds and seedlings respond to mucus cues of the slug *A. subfuscus* highlights the fact that information regarding attack risk has important benefits (88% less herbivory, Fig. 3B) as well as costs (16% less biomass at three weeks, Fig. 2C).

Mucus-exposed seeds germinated faster than control seeds (Fig. 1), suggesting that kairomone-accelerated seed germination may increase the likelihood of avoiding attack and/or decrease attack-related damage. This appears counter-intuitive, since plants that detect herbivory risk might find it more adaptive to delay germination in hopes of avoiding the threat. We believe that the explanation for our results lies in the fact that generalist slugs like *A. subfuscus* avidly consume both seeds and seedlings. While seeds possess fixed resources, photosynthesis-related energetic gain allows seedlings to increase the energy allocable to growth and/or defense. Faster germination may lead to a faster defense response, while this increased allocation of energy to defense may cause the plant to grow smaller. Predator-induced changes in hatching speed have been extensively documented in animals (Horn and Chivers 2021): embryonic exposure to predator cues speeds hatching in both frogs and fish (Chivers et al. 2001; Kusch and Chivers 2004) but reduces it in salamanders (Sih and Moore 1993). Herbivores are known to change germination speed via endozoochory (seed passage through an animal digestive tract; Bilal 2016) or feeding-related scarification of the seed coat (Karban and Lowenberg 1992). Parental experience can also have an effect: herbivory on *Arabidopsis thaliana* plants, for example, can increase jasmonic-acid levels and speed germination in subsequently produced seeds (Singh et al. 2017). Much less is known about the effect of herbivore risk cues on seed germination. The only work similar to ours appears to be a recent study evaluating the potential use of naturally occurring substances in agriculture that dipped pepper (*Capsicum annuum*) seeds in *Arion vulgaris* mucus as a pre-sowing seed treatment to promote faster germination and decreased plant growth (Zolovs et al. 2022). Treated seeds germinated 14% faster than control seeds, a result that they attributed to mucus acting like a sponge to store and hold water around the seed coat. The authors were unable to explain, however, why seedlings emerging from treated seeds were (despite a 1.5-day germination advantage) 24% smaller than control seedlings after 3 weeks (Zolovs et al. 2022). We believe that a risk-mediated trade-off in resource allocation—discussed below—may explain both their results and ours. Follow-up experiments are necessary to determine whether the differences in germination speed that we and Zolovs et al (2022) noted are ‘ecologically relevant’, i.e., do they actually decrease the window of seed/seedling vulnerability.

While kairomone-induced increases in germination speed may allow the plant to defend itself more quickly, such induced responses are likely to come with costs. Our competition assay revealed that seedlings produced by mucus-treated seeds—despite a statistically significant advantage in germination time—weighed 15% less than control plants after three weeks. This result parallels the findings of Zolovs et al (2022) and likely reflects a growth-defense trade-off in which herbivore-threatened plants allocate energy that could otherwise be used for growth toward the production of energetically costly defenses (Huot et al. 2014). This also agrees with previous research showing that tomato (*Solanum lycopersicum*) seeds exposed to snail (*Helix aspersa*) mucus produced seedlings smaller than control plants (Orrock et al. 2018), although a prior experiment that exposed *B. nigra* seeds to *H. aspersa* mucus found no treatment-related differences in the number or length of leaves (Orrock 2013).

We found that the kairomone-induced decrease in *B. nigra* growth was accompanied by an important benefit: cotyledons of seedlings exposed to *A. subfuscus* mucus as both seeds and seedlings experienced 88% less herbivory than control cotyledons. Interestingly, kairomone exposure at the seed stage alone did not similarly reduce herbivory. We hypothesize that the response to the mucus cue diminishes over time, explaining why there appears to be a stronger response of seedlings to mucus than seeds alone. Both results parallel those found in *B. nigra* exposed to *H. aspersa* mucus: seedling herbivory was lower only when kairomones are applied at both the seed and seedling stage (Orrock 2013). Research exposing *S. lycopersicum* seeds and seedlings to *H. aspersa* mucus found that kairomone-mediated induction of polyphenol oxidase, peroxidase, and lipoxygenase reduced herbivore damage (Orrock et al. 2018), and other work demonstrated that treating damaged *Arabidopsis thaliana* with *Arion lusitanicus* slime induced the anti-herbivore jasmonic-acid pathway (Falk et al. 2014).

While this study is unusual in its focus on cotyledons and its suggestion that even these first photosynthetic organs are responsive, there are a few caveats that need to be considered. The most important of these is that we did not identify a potential mechanism for these tradeoffs. Brassicaceous plants rely heavily on glucosinolate-based defenses against herbivory (Hopkins et al. 2009; Ishida et al. 2014), and higher glucosinolate levels decrease mollusk attack in *Arabidopsis* (Falk et al. 2014) and *B. napus* (Giamoustaris and Mithen 2008). Previous work has shown that mucus application does induce defense in large plants (Falk et al. 2014; Meldau et al. 2014; Orrock et al. 2018). While we had originally planned to assess glucosinolates, there was too little foliage on 3-week-old plants to do so; future work should either develop glucosinolate methods for cotyledons or grow plants until large enough to conduct glucosinolate analyses on the true leaves. Additionally, in our choice bioassay, we presented the slugs with cotyledons to assess *A. subfuscus* herbivory preferences. This was necessary because of the small and irregular sizes of the true leaves in our 3-week old plants, but plant-induced defense studies typically assess induced defenses in true leaves (Barton and Koricheva 2010), and although cotyledons and true leaves have differing functions (Chandler 2008), they are homologous organs and similar in terms of development (Kaplan and Cooke 1997). The differences between cotyledons and true leaves may affect the way induced defense is allocated in plants, although previous research has shown that *B. juncea* cotyledons also express glucosinolate-based defense (Wallace and Eigenbrode 2002). Finally, there is the possibility that the observed reduction of *B. nigra* growth reflects a defensive strategy rather than a resource-mediated trade-off between growth and defense. Increases in cell wall toughness may reduce elongation, for example, and the oft-observed association between JA pathway induction and reduced growth in many plant–herbivore systems may not necessarily reflect a cause–effect relationship (Ballare and Austin 2019).

Overall, our research demonstrates that herbivore kairomones can affect both seeds and seedlings in ways that alter both their growth and susceptibility to attack. Slugs are agricultural pests that can cause significant damage to crops (Barua et al. 2021), and climate change is likely to increase both their range and impact on plant communities (Wilson et al. 2015). With mucus cues present in plant communities globally, future studies examining kairomone use in multiple plant species and the persistence of mucus signals in the environment could be useful for understanding significant and unexplained variation in plant defense observed in natural systems.

## Data Availability

All data archived on figshare (https://doi.org/10.6084/m9.figshare.25481752.v1). This will be the permanent repository.
